# Potential metabolic monitoring indicators of suicide attempts in first episode and drug naive young patients with major depressive disorder: a cross-sectional study

**DOI:** 10.1186/s12888-020-02791-x

**Published:** 2020-07-28

**Authors:** Ke Zhao, Siyao Zhou, Xiang Shi, Jianjun Chen, Yaoyao Zhang, Kaili Fan, Xiangyang Zhang, Wei Wang, Wei Tang

**Affiliations:** 1 School of Mental Health, Wenzhou Medical University, Wenzhou, Zhejiang China; 2 CAS Key Laboratory of Mental Health, Institute of Psychology, Chinese Academy of Sciences, Beijing, China; 3 Department of Psychology, University of Chinese Academy of Sciences, Beijing, 100101 China; 4 The Affiliated Kangning Hospital of Wenzhou Medical University, Shengjin Road, Lucheng District, Wenzhou, 325007 Zhejiang Province China; 5 Wenzhou Medical University, Wenzhou, Zhejiang China

**Keywords:** Major depressive disorder, Suicide attempt, Metabolism, Biomarker

## Abstract

**Backgrounds:**

Major depressive disorder is an ordinary mental disorder, and suicide is considered to be a major concern among patients with MDD. Previous studies focused on the relationship between suicide attempts and metabolism in elderly patients with MDD, while ignore the young people. The aim of this study is to find the potential relationship between suicide attempts and metabolism in young patients with MDD to find a way to prevent and ultimately reduce suicide in young patients with MDD.

**Methods:**

Cross-sectional design was employed in the study.740 patients aged between 18 and 45 years old with MDD had been consecutively recruited in this study between 2011 and 2017, 128 of whom had suicide attempts. Their serum samples used to monitor fasting blood glucose, serum lipids as well as socio-demographic characteristics were collected. Besides, some clinical scales were also employed to measure symptoms of anxiety, depression and other conditions.

**Results:**

This study indicated that compared with non-suicide attempters, suicide attempters in young patients with MDD showed higher levels of FBG, TC, LDL-C (all *p* <  0.05) and lower levels of HDL-C(*p* <  0.001). Further logistic regression analysis suggested that suicide attempts were associated with increased FBG, decreased HDL-C, the course of disease, HAMD scores and obvious anxiety.

**Conclusions:**

Suicide attempts in young patients with MDD may be predicted by metabolic levels in the future. And our findings suggested that the level of FBG and HDL-C can be promising biomarkers to predict the occurrence of this event.

## Background

Major depressive disorder (MDD) is a common mental disorder characterized by an insidious onset and recurrent course. It manifests as depressed, reduced interest, impaired concentration, insomnia or even intense suicidal ideation [1]. MDD is recognized as one of the leading causes of burden, which is associated with increased years lived with disability (YLDs) [2]. Suicide is common in MDD and is considered to be a major concern among patients with MDD [3]. Suicidal behavior is a global cause of injury and mortality, with the World Health Organization (WHO) ranking it as the 15th leading cause of death [4]. Thus, it is arguably the most severe consequence of MDD [5].

Earlier studies have indicated that current major episode of depression [6], gender [7], age, education level, chronic diseases [8], the disturbance of metabolism [9], alcohol dependence [10], smoking [11], among others, are risk factors for suicide attempts. However, the exact mechanisms underlying these risk factors remain unknown. According to the data from WHO in 2017, about 800,000 people commit suicide worldwide every year. The incidence of suicide among youth is higher than among other age groups; the WHO defines youth as those aged between 18 and 45 years. Further, rapid growth in the incidence of suicide occurs between the early period of adolescence and young adulthood [12]. Young people are the backbone of a nation; thus, suicidal attempts among youth warrant social concerns.

Research suggests that metabolic levels have a bidirectional relationship with psychological distress [13] and are also associated with suicide attempts. The relationship between metabolism and suicide attempts has been discussed in many studies. For instance, a community-based cohort study reported that metabolic abnormalities were correlated with suicide [14]. The sample for this study was drawn from the community and all participants were greater than 30 years of age; none had specific mental health diseases. It could be better if they also focused on other different health care systems or more specific people. Another study reported that lower serum cholesterol and triglyceride levels were in men with the bipolar disorder, who had attempted suicide relative to those who had not attempted suicide [9]. However, another study of patients with bipolar disorder aged between 21 and 60 years found that the incidence of suicide attempts was associated with higher cholesterol levels [15]. Thus, both studies examined the correlation between serum cholesterol and suicide among bipolar disorder patients, but each study came to a different conclusion. It should be noted that several other studies have reported that low cholesterol increases suicide risk [16–18]. Nonetheless, this association requires further investigation. Research also suggests that blood glucose levels are associated with suicide risk. A pilot study suggested that abnormal glucose metabolism may reflect biological changes prior to or concomitant with suicide [19]. Diabetes mellitus among patients with depression is also associated with a higher rate of suicide attempts [20]. What’s more, a cohort study about investigating the risk factors for future suicide found that diabetes rather than increased blood glucose was associated with suicide [21]. However, due to the low incidence of metabolic diseases in young people, most studies have neglected to study this group. Further, we speculate that abnormal metabolism in patients with depression, although not up to the disease standard, may also have an impact on suicidal behavior [22].

When considering the published literature, it is clear that there are few studies that have investigated the relationship between metabolic levels and suicide attempts in patients with MDD, and among the available studies, few have studied youth. For example, one study of patients with MDD found that serum lipid levels were associated with suicide attempts in adults [23]. Another found that glucose disturbances and lipid metabolism disturbances commonly exist in patients with depression aged 35 years and older [24].Since of all the age groups, the incidence of suicide in youth is higher. However, most related studies either concentrated on the whole crowd nor on the middle-aged and elderly, while ignoring the group of youth. The group of youth is worthy of attention, for they are pillars of the society. Further, due to life pressures and other factors, an increasing number of young people experience depression; some ignore it at first and it can then develop into MDD with an increased risk of suicide. Given the potential links between metabolic levels and suicide attempts in adults with MDD, we speculated that the relationship between metabolic levels and suicide attempts in young patients with MDD can also be found. Specifically, we speculated that changes in blood glucose and cholesterol levels may directly or indirectly influence the occurrence of suicide attempts in young patients with MDD.

Therefore, in the current study, we systematically analyzed the correlations between metabolic levels and suicide attempts in patients with MDD aged 18–45 years. It was hoped that these results would contribute to the identification of biomarkers for suicide ideation in MDD so that early intervention can be achieved.

## Methods

### Study design

A cross-sectional design was employed in this study. The aim of this study was to identify if there is a relationship between suicide attempts and metabolism in young patients with MDD, with the goal of effectively preventing and ultimately reducing the incidence of suicide in young patients with MDD.

### Subjects

In total, 740 patients with MDD, who met the inclusion and exclusion criteria, were consecutively recruited to this study between 2011 and 2017; 128 of the patients had attempted suicide previously. The study inclusion criteria were: (1) Han ethnicity, aged between 18 and 45 years; (2) conformed diagnosis of MDD based on the DSM-IV; (3) score of 24 or greater on the 17-item Hamilton Rating Scale for Depression (HAMD) and patients experiencing their first episode of depression at the time of enrolment in the study; (4) no previous use of antidepressants or antipsychotics. The exclusion criteria were: (1) psychiatric diagnosis other than MDD; (2) patient is labile or has a severe physical condition, for example, epilepsy, liver or kidney diseases, diabetes, or heart disease; (3) patient is pregnant or breastfeeding; (4) patient has a drug addiction or alcohol dependence; (5) patient refuses to participate. Patients who agreed to participate were required to give a detailed medical history and then underwent a systematic physical examination as well as laboratory testing.

### Collection and evaluation of socio-demographic and clinical measures

The socio-demographic data included age, course of disease, age at onset, gender, cultural degree, marital status, and body mass index (BMI). Further, for patients with MDD who had previous suicide attempts, detailed information about these attempts was collected.

The 17-item HAMD was used to quantify each patient’s severity of depression. The HAMD ratings were confirmed by semi-structured clinical interviews with a maximum score of 52 [25]. The standard for evaluation was as follows: ≤7, not depressed; ≥8, depressed; ≤17, mild to moderate depressive symptom; and ≥ 24, severe depression [26]. The cut-point of 24 was used to distinguish the presence or absence of MDD.

In addition, the Hamilton anxiety rating scale (HAMA) was employed to comprehensively measure the severity of perceived anxiety symptoms. Items on this measure are rated on a 5-point scale from 0 (not present) to 4 (severe). The maximum total HAMA score is 56 [27]. If the total score reaches or exceeds 21, the patient is considered to have obvious anxiety symptoms.

The Positive and Negative Syndrome Scale (PANSS) was employed to find whether there is a connection between suicide attempt and psychotic symptom in patients with MDD or not, because the independent increase of positive symptoms of psychosis as well as the symptoms of depression can lead to the increase of suicidal ideation [28, 29]. Each item is scored on a 7-point scale based on the level of psychopathology. In this study, patients with a total positive symptom subscale score reaching or exceeding 15 were defined as having psychotic symptoms [29, 30].

The Mini-International Neuropsychiatric Interview (MINI) was used to ensure the accuracy and consistency of the diagnostic process. The MINI comprises modules for 17 psychiatric diagnoses. Each item is answered either “yes” or “no”. In order to facilitate responses, examples are provided for each question.

### Requests of the staff

At least two qualified psychiatrists who had received good training on the use of the HAMD, HAMA, and PANSS scales were needed for this study. After staff training, the inter-rater correlation coefficient for each scale was greater than 0.8. All interviewers were completely unaware of the patient’s condition before the interview process.

### Measurement of suicide attempts

Suicide attempt was defined as the presence of suicidal thoughts or an attempt at suicide, but for various reasons, suicide was not successful or complete. Each patient was carefully asked whether they had attempted suicide in their lifetime; if they answered yes, they were categorized as a suicide attempter. Then, suicide attempters were asked further questions to determine the frequency, approach, and date of their suicide attempts. If they answered uncertainly or unclearly, complementary information was gained by asking their family members, relatives, or friends. In this study, suicide attempts in patients with MDD were related to this depressive disorder.

### Collection and detection of serum samples and others

Overnight fasting blood samples of the patients were collected by the nurses and then delivered to the hospital’s laboratory center to be measured by a chemiluminescence immunoassay using Cobas E610 (Roche, Basel, Switzerland). The serum indices measured included: fasting blood-glucose (FBG), total cholesterol (TC), high-density lipoprotein cholesterol (HDL-C), low-density lipoprotein cholesterol (LDL-C) and triglyceride (TG). The normal range for each index was 3.90–6.10 mmol/L for FBG, less than 5.17 mmol/L for TC, 1.04–2.07 mmol/L for HDL-C, less than 3.10 mmol/L for LDL-C, and 0.56–1.69 mmol/L for TG. Further, systolic blood pressure (SBP) and diastolic blood pressure (DBP) were also monitored.

### Statistical analysis

Socio-demographic data and clinical characteristics were compared between participants with and without suicide attempts with the use of one-way analysis of variance (ANOVA) and the Chi-squared test. In addition, binary logistic regression analysis was utilized to analyze the factors associated with suicide attempts in young patients with MDD. Odds ratios (ORs) and 95% confidence intervals (95% CIs) were evaluated. The dependent variable was whether or not the patient with MDD had attempted suicide and the independent variables were age, course of disease, gender, cultural degree, marital status, HAMD score, psychotic symptoms, obvious anxiety, FBG, TC, HDL-C, TG, and LDL-C. Then Pearson correlation analysis was used to explore the potential correlations between the serum indicators and clinical variables. Bonferroni corrections were used to adjust for multiple testing. All tests were two-sided with a *p* value of 0.05. Correlations were quantified and assessed by coefficient values, and the effect size. Data analysis was conducted by using SPSS version 25.0 (SPSS Inc., Chicago, IL).

## Results

Table 1 compares the socio-demographics, clinical characteristics, and metabolic indices of patients with MDD who had attempted suicide with those who had not. In total, 128 of the 740 (17.3%) patients with MDD had attempted suicide while the remaining 612 patients (82.7%) were classified as non-suicide attempters. Compared with non-suicide attempters, suicide attempters had relatively higher serum levels of FBG, TC, and LDL-C, and lower HDL-C levels (all *p* <  0.05). Further, young patients with MDD who had attempted suicide also showed differences in the course of the disease, HAMD scores, HAMA scores, PANSS scores, and blood pressure compared with those who had not attempted suicide (all *p* <  0.05).

**Table 1:** Socio-demographics, clinical characteristics and metabolic indictors between patients with and without suicide attempts

Variable	MDD without suicide attempt (*n* = 612)		MDD with suicide attempt (*n* = 128)		F/X^2^	*P* value
Age, mean (SD), y	22.68	4.09	22.98	3.765	0.610	0.435
Course of disease, mean (SD), m	4.58	2.99	5.54	3.85	9.710	0.002**
Gender						
Male, n (%)	236	38.6	51	39.8		
Female, n (%)	376	61.4	77	60.2		
Cultural degree						
Junior high school education level, n (%)	29	4.8	13	10.2		
Senior high school education level, n (%)	300	49.0	55	43.0		
University education level, n (%)	240	39.2	50	39.0		
Postgraduate education level, n (%)	43	7.0	10	7.8		
Marital status						
Single	384	62.7	86	67.2		
Married	228	37.3	42	32.8		
HAMD, mean (SD)	29.71	2.84	32.08	2.75	74.600	< 0.001 ***
HAMA, mean (SD)	20.03	3.10	23.20	3.47	105.711	< 0.001 ***
Obvious anxiety (HAMA ≥21), n (%)	270	44.1	102	79.7	740	< 0.001 ***
Psychotic symptoms (PANSS ≥15), n (%)	43	7.02	24	18.75	6.643	0.012*
BMI, mean (SD), kg/m^2^	24.31	1.87	24.14	2.75	0.694	0.405
SBP, mean (SD), mmHg	111.82	9.19	119.42	12.57	62.909	< 0.001***
DBP, mean (SD), mmHg	72.98	5.81	76.63	7.01	38.651	< 0.001***
FBG, mean (SD), mmol/L	5.32	0.61	5.61	0.70	23.145	< 0.001***
Increase (FBG > 6.10 mmol/L), n (%)	66	10.8	29	22.7		
Normal (3.90–6.10 mmol/L), n (%)	546	89.2	99	77.3		
TC, mean (SD), mmol/L	5.00	1.07	5.80	1.14	57.461	< 0.001***
Increase (TC ≥ 5.17 mmol/L), n (%)	255	41.6	83	64.8		
Normal (TC < 5.17 mmol/L), n (%)	357	58.4	45	35.2		
HDL-C, mean (SD), mmol/L	1.25	0.28	1.12	0.31	20.920	< 0.001***
Decrease (HDL-C < 1.04 mmol/L), n (%)	147	24.0	55	43.0		
Normal (1.04–2.07 mmol/L), n (%)	465	76.0	73	57.0		
LDL-C, mean (SD), mmol/L	2.84	0.86	3.17	1.01	14.378	< 0.001***
Increase (LDL-C ≥ 3.10 mmol/L), n (%)	222	36.3	70	54.7		
Normal (LDL-C < 3.10 mmol/L), n (%)	390	63.7	58	45.3		
TG, mean (SD), mmol/L	2.12	0.97	2.25	1.05	1.756	0.185
Increase (TG > 1.69 mmol/L), n (%)	368	60.0	79	61.7		
Normal (0.56–1.69 mmol/L), n (%)	244	40.0	49	38.3		

Note: *MDD* major depressive disorder, *HAMD* Hamilton depression rating scale, *HAMA* Hamilton anxiety rating scale, *PANSS* The Positive and Negative Syndrome Scale, *BMI* body mass index, *SBP* systolic blood pressure, *DBP* diastolic blood pressure, *FBG* fasting blood glucose, *TC* total cholesterol, *HDL-C* high-density lipoprotein cholesterol, *LDL-C* low-density lipoprotein cholesterol, *TG* triglyceride. *p <  0.05; **p <  0.01; ***p <  0.001

Further binary logistic regression analysis(Table 2) indicated that in young patients with MDD, suicide attempts were related to the course of disease, with an OR of 1.111 (95% CI: 1.043–1.184; df = 1, *p* <  0.01), HAMD scores, with an OR of 1.217 (95% CI: 1.105–1.341; df = 1, *p* <  0.001), obvious anxiety, with an OR of 3.050 (95% CI: 1.806–5.149; df = 1, *p* <  0.001), increased FBG, with an OR of 1.918 (95% CI: 1.104–3.331; df = 1, *p* <  0.05), and decreased HDL-C, with an OR of 1.794 (95% CI: 1.127–2.857; df = 1, *p* <  0.05).

**Table 2:** Logistic regression for factors associated with suicide attempts in patients with MDD

Variable	B	Odds Ratio	95% Confidence Interval		*P* value
			Lower	Upper	
Age	0.054	1.055	0.977	1.140	0.172
Course of disease	0.105	1.111	1.043	1.184	0.001**
Cultural degree	−0.227	0.797	0.584	1.087	0.152
Gender	−0.230	0.795	0.515	1.227	0.300
Marital status	−0.506	0.603	0.321	1.131	0.115
The scores of HAMD	0.197	1.217	1.105	1.341	< 0.001***
Psychotic symptom	0.080	1.083	0.569	2.061	0.808
Obvious anxiety	1.115	3.050	1.806	5.149	< 0.001***
FBG increase	0.651	1.918	1.104	3.331	0.021 *
TC increase	−0.028	0.972	0.566	1.669	0.918
HDL-C decrease	0.585	1.794	1.127	2.857	0.014 *
TG increase	−0.440	0.644	0.409	1.013	0.057
LDL-C increase	0.198	1.219	0.754	1.970	0.419

Note: *HAMD* Hamilton depression rating scale, *FBG* fasting blood glucose, *TC* total cholesterol, *HDL-C* high-density lipoprotein cholesterol, *TG* triglyceride, *LDL-C* low-density lipoprotein cholesterol. * *p* <  0.05; **p <  0.01; ***p <  0.001

Pearson correlation analysis revealed significant correlations between FBG levels and the following parameters: depression (*r* = 0.282, df = 740, *p* <  0.001), positive symptoms (*r* = 0.149, df = 740, *p* <  0.001), and anxiety (*r* = 0.091, df = 740, *p* = 0.013). There were also significant correlations between TC and depression (*r* = 0.563, df = 740, *p* <  0.001), positive symptoms (*r* = 0.215, df = 740, *p* <  0.001), and anxiety (*r* = 0.277, df = 740, *p* <  0.001). Further, LDL-C was significantly correlated with depression (*r* = 0.365, df = 740, *p* <  0.001), positive symptoms (*r* = 0.122, df = 740, *p* <  0.001), and anxiety (*r* = 0.193, df = 740, *p* <  0.001). Finally, HDL-C was significantly associated with depression (*r* = − 0.168, df = 740, *p* <  0.001), positive symptoms (*r* = − 0.117, df = 740, *p* = 0.001), and anxiety (*r* = − 0.086, df = 740, *p* = 0.019) (Table 3). Aside from the significant correlations between level of FBG and anxiety (*p* = 0.013) and between level of LDL-C and anxiety (*p* = 0.019), all significant differences passed Bonferroni correction (Bonferroni corrected *p* <  0.05/12 = 0.0042).

**Table 3:** Inter-corrections between some serum variables and clinical variables

Variables	Depression		Positive symptom		Anxiety	
	r	p	r	p	r	p
FBG	0.282	< 0.001*	0.149	< 0.001*	0.091	0.013
TC	0.563	< 0.001*	0.215	< 0.001*	0.277	< 0.001*
HDL-C	−0.168	< 0.001*	−0.117	0.001 *	−0.086	0.019
LDL-C	0.365	< 0.001*	0.122	0.001 *	−0.193	< 0.001 *

Note: *FBG* fasting blood glucose, *TC* total cholesterol, *HDL-C* high-density lipoprotein cholesterol, *LDL-C* low-density lipoprotein cholesterol. * Bonferroni corrected *p* < 0.05/12 = 0.0042

## Discussion

To the best of our knowledge, this study is the first to focus on exploring the relationship between metabolic levels and suicide attempts among young (aged 18–45 years), first episode, drug-naive patients with MDD. The sample size of this study was relatively large. The study aimed to examine potential relationships between suicide attempts and metabolic levels in young patients with MDD to identify a promising way to effectively prevent and ultimately reduce the incidence of suicide in young patients with MDD.

In general, this study found that, among young patients with MDD, suicide attempts were associated with relatively high levels of FBG, TC, and LDL-C in serum, low levels of HDL-C, and high blood pressure readings; these indices may be promising biomarkers for evaluating the risk of suicide attempts in patients with MDD. Binary logistic regression analysis suggested that course of the disease, HAMD score, elevated FBG level, decreased HDL-C level, and obvious anxiety were independent risk factors for suicide attempts in young patients with MDD.

In general, the course of the disease and HAMD scores can reflect the severity of MDD, which may predict the occurrence of suicide attempts in patients. Further, the findings of the current study suggest that high serum FBG levels are associated with suicide risk in patients with MDD aged from 18 to 45, which is in accordance with previous studies, to a certain extent. Koponen found higher levels of blood glucose in the oral glucose tolerance test (OGTT) at baseline and 2 h in patients with depression who had attempted suicide compared to those without suicide attempts [24]. Other studies also reported the relationship between high level of blood glucose and suicide. Batty et al. conducted a 14-year follow up study and found that increased fasting blood glucose increased the risk of suicide [21]. Further, Ko et al. reported that elevated levels of fasting plasma glucose may lead to suicidal risk [31]. Glucose is a ubiquitous source of energy and provides energy to human brain cells. Some psychological processes, such as self-control and regulation of emotions, are associated with the supply of glucose in brain cells [32]. Elevated peripheral blood glucose can reflect intracellular glucose deficiency. When brain cells lack glucose, the dysregulation of emotions may occur, which can lead to aggressive impulses, pessimism and impulsivity. Hyperglycemia is a decrease in the ability of cells to absorb circulating glucose due to insulin deficiency or insulin resistance. This may explain why Diabetes mellitus combined with depression showed higher past rate of suicide attempts. Morbid blood glucose metabolism can greatly aggravate the risk of suicide attempts. Thus, we speculate that fluctuant blood glucose in the normal range, particularly elevated blood glucose, influences the uptake of glucose in brain cells, which can also increase the risk of suicide attempts. Moreover, we found correlations between FBG levels and depression, anxiety, and psychotic symptoms. Suicidal ideation can be further amplified when depression worsens [33]. Furthermore, Wanqiu Yang et al. suggested that anxiety deteriorated the depression and in patients with MDD increased the rate of suicide attempts [34]. Elevated glucose levels are associated with mood disorders [35]. Thus, we speculate that changes in FBG, especially increases in FBG, can also indirectly increase the risk of suicide attempts by aggravating the degree of anxiety, depression symptoms, and psychotic symptoms.

Our study also revealed that patients with MDD, aged 18–45 years, who had attempted suicide had higher TC and LDL-C levels than non-attempters. This is in contrast to the findings of some studies. For example, in a study of patients in Northern Mexico, Segoviano-Mendoza et al. found that patients with MDD who had attempted suicide had lower levels of cholesterol compared with healthy people [18].The difference may occurred by the regional disparities and the ways of comparison was different from us. What’s more, Papadopoulou et al. suggested that psychiatric patients who were used to have suicidal attempts showed lower TC than normal people [36]. However, the sample size of this study was relatively small and the sample was comprised of patients with heterogeneous psychiatric disorders. Interestingly, Bartoli et al. found that serum cholesterol was not associated with suicide attempts [16]. The participants in this study included patients who had taken antipsychotics and / or antidepressants before, and drugs can have an effect on serum cholesterol levels as well as lead to confounding factors of the results. Besides, the average age of the patients in the above study was relatively high. However, some other studies drew another conclusion. For instance, Brunner et al. suggested that serum cholesterol and suicide had a positive association [37]. According to 2010 Korean National Health and Nutrition Examination Survey, Sun-Mi Kim et al. found that people with suicidal ideation had relatively higher TC than those without suicidal ideation [38]. Nevertheless, this study put the emphasis on the relationship between metabolic syndrome and suicidal ideation in adolescents and adults, without specific reference to patients with MDD. Even though, we can also get inspired that the level of cholesterol may be different in different groups or conditions.

Our study focused on young people based on the premise that, due to the modern lifestyle and improvements in living standards, an increasing number of young people are susceptible to dietary or other factors that may influence serum lipid levels. Besides, previous studies have focused on western samples, while the current study was conducted with a Chinese sample. These factors may underlie the variation in findings observed here relative to the published literature. In short, the relationship between suicide attempts and both TC and LDL-C levels remain controversial to some extent; however, it may be possible to use level of HDL-C to predict suicide attempts in young patients with MDD based on our finding that decreased HDL-C level was a significant risk factor of suicide attempts in young patients with MDD. We also found relationships between HDL-C and anxiety, depression, and psychotic symptoms. Previous studies reported that the increase of positive symptoms as well as the symptoms of depression can increase the risk of suicidal ideation [28, 29]. Further, Capron et al. reported that anxiety can amplify the stress response, which in turn can increase the suicidal tendency [33]. What’s more, Buydens-Branchey et al. reported that low HDL-C was associated with reduced 5-HT function [39]. When 5-HT function reduced, it can aggravate the degree of depression and increase the risks of suicide behaviors. So, we speculated that clinical states may take part in the potential link between HDL-C and suicide attempts. The result is also similar with other previous studies, for example, Zhang J et al. found that low level of HDL-C was associated with suicide attempt in young healthy women. Although the participants didn’t have depression, the results may inspire us that the connection between HDL-C and suicide attempt is direct [40]. Further, Maes et al. made a research and found that depressed men with suicide attempt had a significantly lower level of HDL-C than those without suicide attempt [41]. As for the potential mechanisms, there are still no final conclusions and there are few studies on the relationship between HDL-C and suicide attempt in patients with MDD. Maybe some related personality traits or perceived stress are potential mediators for such link. Firstly, personality traits can affect emotional perception, decision-making, and then behavior [42]. For example, Peters et al. reported that impulsivity was associated with suicide attempt [43]. And low serum cholesterol has something to do with impulsivity [44]. So, we thought that impulsive personality may play a role between HDL-C and suicide attempt. Even though, further studies are still necessary to find the potential mechanism. What’s more, referring to perceived stress, Shelef et al. reported that perceived stress was identified as one of primary determinants among young suicide attempters [45]. And Gowey MA reported that increased depressive symptoms and perceived stress are usually associated with decreased level of HDL [46]. So, we considered that a certain degree of perceived stress may take part in the influence of lower HDL-C on suicide attempts.

The current study did have several limitations that should be considered. Firstly, the cross-sectional design does not allow for the determination of causal relationships between metabolic levels and suicide attempts in young patients with MDD. Therefore, a longitudinal design should be considered in future studies in order to study causal relationships. Secondly, several socio-demographic factors that may influence the results were not included in the current study. For instance, alcohol consumption, cigarette smoking, and economic conditions. Thirdly, the current study was restricted to a certain age group. Future studies should include a larger age range.

## Conclusions

Identifying strong and accurate risk factors is a promising way to effectively prevent and ultimately reduce the incidence of suicide in young patients with MDD. The findings of the current study revealed that, in young patients with MDD with suicide attempts, levels of metabolic indicators were quite different from those of young MDD patients without suicide attempts. Among the metabolic indicators, we found that levels of FBG and HDL-C were promising biomarkers; in particular, elevated FBG levels and decreased HDL-C levels. The results are important for clinical practice as changes in serum indicators or psychiatric symptoms may predict a certain risk of suicide attempts in young patients with MDD. Nonetheless, further studies are still needed to clarify the intrinsic relationships between metabolism and suicide attempts in young patients with MDD, as the results of the current study were inconsistent with previous studies in some instances. There are several potential factors that could account for these differences; these require further study in the future.

## Data Availability

Data can be gained from the corresponding author.

## Abbreviations

MDD: Major Depressive Disorder
YLD: Years Lived with Disability
HAMD: Hamilton Depression Rating Scale
HAMA: Hamilton Anxiety Rating Scale
PANSS: The Positive and Negative Syndrome Scale
BMI: Body Mass Index
SBP: Systolic Blood Pressure
DBP: Diastolic Blood Pressure
FBG: Fasting Blood Glucose
TC: Total Cholesterol
HDL-C: High-density Lipoprotein Cholesterol
LDL-C: Low-density Lipoprotein Cholesterol
TG: Triglyceride
